# Ligand Influence on CuInS_2_ Quantum Dot Photoconductive Films

**DOI:** 10.3390/nano16040258

**Published:** 2026-02-16

**Authors:** Yizun Wang, Hrilina Ghosh, Siva Sivoththaman

**Affiliations:** Department of Electrical and Computer Engineering, University of Waterloo, Waterloo, ON N2L 3G1, Canada; y2735wang@uwaterloo.ca (Y.W.); sivoththaman@uwaterloo.ca (S.S.)

**Keywords:** quantum dot, ligand exchange, photoluminescence, photodetection

## Abstract

In this work, we investigate the effect of ligand chemistry on the optical and electrical properties of copper indium disulphide (CuInS_2_) quantum dots (QDs) and evaluate their suitability for photodetection with simple device structures. CuInS_2_ QDs capped with dodecanethiol (DDT) ligands were synthesized, followed by processes to exchange the DDT with thioglycolic acid (TGA), mercaptopropionic acid (MPA), or thioacetamide (TAA) ligands. Photoluminescence (PL) and UV-Visible absorption studies revealed that while TGA- and MPA-capped QDs retained strong emission, TAA-capped QDs exhibited significant quenching, indicating surface defect formation due to poor ligand binding. Metal–semiconductor–metal (MSM) test structures were fabricated using the QD films as the active layer to study their electrical properties under dark and UV-illuminated conditions. Devices based on MPA- and TGA-capped QD films demonstrated currents that were 7–9 times higher than those of devices with native DDT ligands, with significantly enhanced photocurrent-to-dark current ratios of 2.6 and 1.7, respectively, highlighting the effective charge transport pathways enabled by the shorter ligands. The device with TGA-capped QD film also responded well to 20 kHz pulsed UV excitation, underscoring the strong potential of this simple MSM structure for photodetection and optical switching applications.

## 1. Introduction

Photodetectors of various types and complexities are being increasingly used across a broad range of applications such as high-resolution imaging [1], optical communications [2], healthcare monitoring [3], high-speed photothermal detection [4], environmental monitoring [5], and remote sensing [6]. Traditionally, silicon-based photodetectors have been known for reliable performance in the visible to near-infrared range [7]. However, the intrinsic constraints and limitations of conventional Si photodetectors in accurate ultraviolet (UV) light detection have prompted the exploration of alternative semiconductor materials with broader spectral sensitivity [8].

Quantum dots (QDs), particularly those synthesized via colloidal synthesis methods, have garnered significant attention for next-generation optoelectronic devices. Owing to their quantum confinement effects, QDs offer tunable bandgaps that span the UV, visible, and infrared (IR) spectral regions [9,10,11]. These unique properties make them suitable for diverse applications, including photodetectors [12,13], solar cells [13,14,15,16], bioimaging [17], and light-emitting diodes [18,19]. Colloidal quantum dots (CQDs), synthesized through solution-based processes, are particularly appealing due to facile surface modification and compatibility with low-cost, scalable device fabrication techniques [20].

Among various CQDs, II–VI and IV–VI group materials such as Cd- and Pb-based QDs have been extensively studied, offering high detectivity, efficient light absorption, and wide bandgap tunability [21,22,23]. Devices based on these QD materials have demonstrated excellent performance in both photodetection and photovoltaic energy conversion. However, the environmental and toxicity issues associated with heavy metals have raised some concern, particularly considering the use of toxic nanomaterials in future, large-scale manufacturing settings [24,25,26]. QDs based on non-toxic materials are also being intensively researched for several application areas, including optoelectronics [27,28,29]. I–III–VI QDs, such as CuInS_2_, CuInSe_2_, AgInS_2_, and AgInSe_2_, are among the promising candidates [30]. These materials not only exhibit reduced toxicity but also offer wide applicability across multiple device architectures.

In optoelectronic device structures that employ QD films, not only the core structure of the QDs but also their surface ligands play a crucial role in charge transport [31] by influencing carrier hopping rate and tunneling barrier. A long-chain surface ligand, such as 1-dodecanethiol (DDT), passivates surface states and stabilizes the QDs. However, for device integration, particularly in photodetectors, these long-chain, non-polar ligands present significant drawbacks. They exhibit weak surface adhesion and impede inter-dot carrier transport, reducing overall device performance. Post-synthesis ligand engineering plays a pivotal role in this regard. Replacing insulating long-chain ligands with shorter, polar alternatives can significantly enhance charge transport across the QD film. As such, the selection and manipulation of QD surface ligands are important in high-performance QD assemblies. I–III–VI QD-based high-performance optoelectronic devices generally rely on core/shell nanostructures, heterojunctions, or complex device architectures to achieve stability and efficiency. In contrast, this work highlights the role of ligand engineering in enabling reproducible photoconductive response from core-only low toxicity CuInS_2_ QD films in simple lateral MSM test arrays.

In this work, we first synthesized colloidal CuInS_2_ QDs, dispersed in hexane, following a low-reaction-temperature regime process [32]. The synthesis process necessarily results in the QDs being stabilized and capped with long-chain 1-dodecanethiol (DDT, 12-carbon chain) ligands. In order to improve the ligand-mediated charge transport characteristics, we performed post-synthesis ligand exchange using three different short-chain ligands: 3-mercaptopropionic acid (MPA, 3-carbon chain), thioglycolic acid (TGA, 2-carbon chain), and thioacetic acid (TAA, 2-carbon chain). Fourier transform infrared spectroscopy (FTIR) and X-ray photoelectron spectroscopy (XPS) were used to analyze the ligand-exchanged QDs and to confirm the successful replacement of the native DDT ligands. Photoluminescence (PL) spectroscopy was also employed to assess the optical properties of the QDs before and after the ligand exchange. To evaluate the impact of ligand exchange on charge transport and optical response, simple lateral metal–semiconductor–metal (MSM) structures were fabricated, with the drop-cast QD film functioning as the conductor layer between interdigitated electrodes. Steady-state current-voltage measurements revealed enhanced conductivity, both in the dark and under UV illumination, for films of QDs capped with short-chain ligands. The ligand-exchanged film also responded well to a frequency-modulated UV signal, underscoring the high potential of the MSM structures for use in switching and photodetection modes.

## 2. Materials and Methods

### 2.1. Synthesis of CuInS_2_ Quantum Dots

CuInS_2_ QDs have been synthesized in a non-injection, moderate-temperature, solution-phase process using copper iodide (CuI) and indium acetate (In(Ac)_3_) as precursors in DDT solvent. A ratio of 1:2 for Cu:In is used at the precursor level. Specifically, 1 mmol of CuI, 2 mmol of In(Ac)_3_, and 5 mL of DDT are introduced into a three-neck round-bottom flask. The reaction flask is purged with nitrogen gas for 10 min while a vacuum line continuously removes the nitrogen to maintain a sterile internal environment.

Once the purging is complete, the temperature is raised to 80 °C using a PID-controlled heating mantle, while the mixture is continuously stirred at 30% of the maximum stirrer speed. The solution is maintained at this temperature until it becomes homogenous, indicating complete dissolution of the precursors. At this stage, the solution should appear yellow. The temperature is then gradually increased in 10 °C increments every 5–10 min, depending on the solution’s consistency. This step is critical because it governs the nucleation and growth of monodisperse QDs. The gradual change in the solution’s color is indicative of the ongoing growth process. As the reaction progresses, the solution undergoes a series of color transitions–yellow, golden, orange, candy red, red, deep red, and finally a viscous, inky burgundy. This final color change signifies the completion of QD growth. The heating mantle is then switched off, and hexane is injected into the reaction flask to dissolve the QDs. The resulting colloidal QD solution is extracted using a syringe.

To purify the as-synthesized QDs, acetone (or ethanol) is added to the QD–hexane solution at a volume ratio of approximately 8:1. The mixture is then centrifuged at 8000 rpm for 5 min, resulting in the precipitation of QDs. The supernatant is carefully decanted, and the precipitate is re-dispersed in fresh hexane. This washing process is repeated five times to completely remove unreacted precursors and excess ligands. The final product consists of purified CuInS_2_ QDs stabilized with DDT ligands (CuInS_2_-DDT) and dispersed in hexane, ready for further processing or characterization.

### 2.2. Ligand Exchange

**Figure 1:**
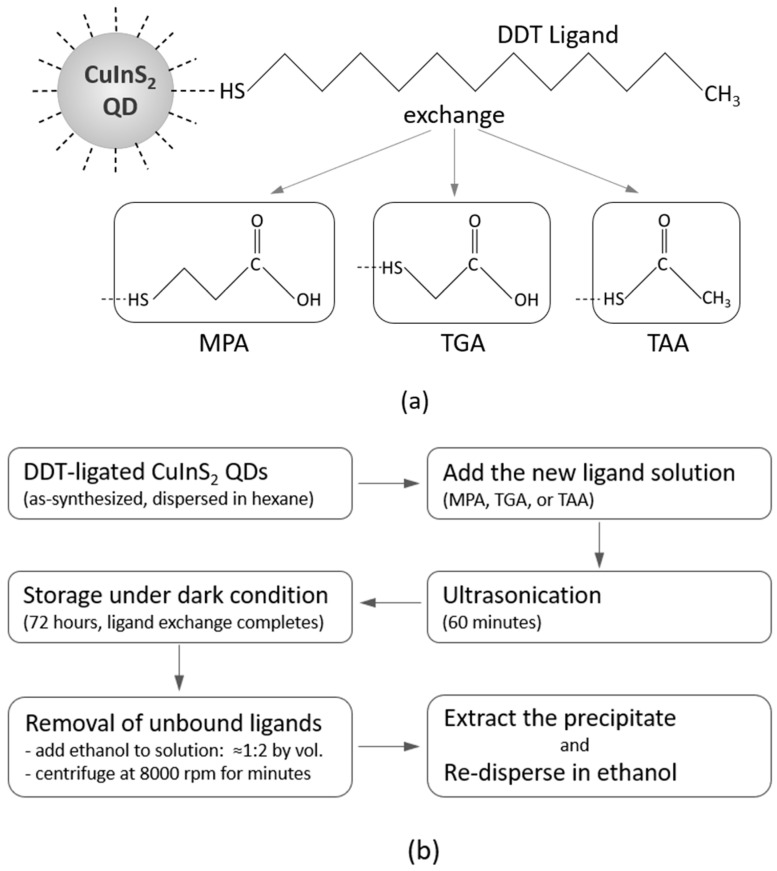
(**a**) Structure of the DDT ligand (in the as-synthesized QDs), and the MPA, TGA, and TAA ligands used for the exchange. (**b**) The generic process flow used for the ligand exchange.

We have explored the use of three short-chain, alkyl thiol (R-SH)-based ligands, MPA (COOH(CH_2_)_2_SH), TGA (COOH(CH_2_)SH), and TAA (CH_3_COSH), to replace the long-chain DDT ligands on the synthesized CuInS_2_ QDs. These short-chain ligands share functional groups similar to those of DDT (CH_3_(CH_2_)_11_SH) but offer enhanced charge transport and improved film adhesion due to their shorter carbon chains. Moreover, unlike DDT, these ligands are polar due to the carboxylic acid group. The ligand structures are depicted in Figure 1a.

The ligand solution (MPA, TGA, or TAA) is first prepared and added to a vial containing the CuInS_2_-DDT QDs. The mixture is ultrasonicated for 60 min to ensure complete dispersion of the QDs, as mechanical shaking alone is insufficient to break up aggregates and achieve thorough mixing. Once the QDs are fully dispersed in the ligand solution, the vial is stored in a dark environment for 72 h to allow complete ligand exchange. After the exchange, the same purification steps described in the previous section are repeated to remove unbound ligands. The precipitated QDs are then collected, dried, and weighed. Finally, for subsequent processing and characterization, the CuInS_2_ QDs capped with the new ligands are redispersed in ethanol. Among the three ligand types tested, the TAA-capped QD solution exhibited a somewhat inferior monodispersity compared to the other two. The generic ligand exchange protocol used is schematically shown in Figure 1b.

While the exchange mechanisms are rather complex and depend on the ligands and their types (X-, L-, Z-) as well as the host surface, the key processes involved are diffusion of the non-native (i.e., replacing) ligand to the QD surface, desorption of the native ligand, and adsorption and rearrangement of the non-native ligand accompanied by surface modifications of the QD [33].

## 3. Results and Discussion

### 3.1. Effectiveness of the Ligand Exchange Process

**Figure 2:**
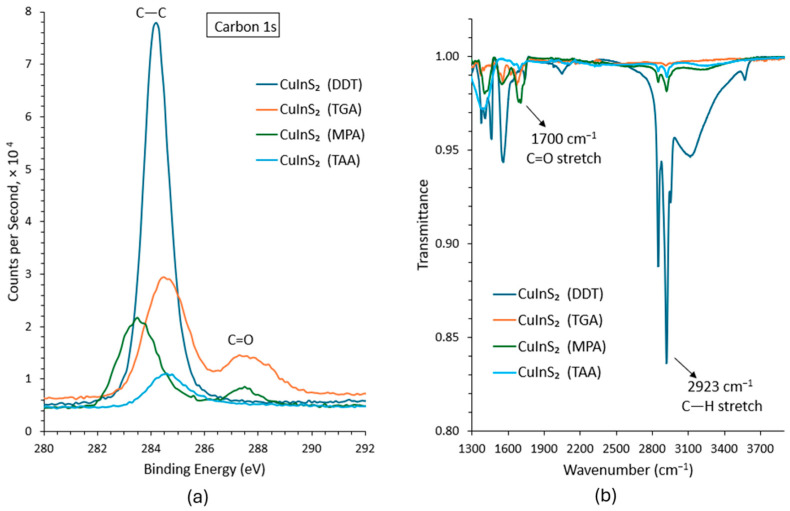
(**a**) Carbon 1s XPS spectra, and (**b**) FTIR spectra, of the synthesized CuInS_2_ QDs capped with DDT, TGA, MPA, and TAA ligands.

The chemical composition of the as-synthesized and ligand-exchanged CuInS_2_ QDs were studied using X-ray photoelectron spectroscopy (XPS) (VG ESCALab 250, Thermo Fisher, Waltham, MA, USA). The carbon 1s XPS spectra of the different ligand-terminated CuInS_2_ QDs are presented in Figure 2a. From the figure, it is observed that a peak corresponding to C=O (≈287 eV) appears for the shorter-chain ligands, further confirming the successful replacement of long alkyl chains (DDT) with shorter ligands by the exchange process. The absence of a distinct C=O peak for the TAA-treated sample can be attributed to the unsuccessful ligand exchange for TAA-capped CuInS_2_ QDs. Unlike MPA and TGA, TAA does not contain a –COOH group, and the ligand exchange resulted in a poorly dispersed, non-colloidal cloudy QD solution. As the TAA ligand exchange was not successful, the resulting sample used for XPS did not have enough TAA ligands to produce the C=O peak.

It is also observed that the intensity of the C 1s peak corresponding to C–C bonds (≈284–285 eV binding energy) diminishes for QDs with shorter-chain ligands. The slight shift of ~1 eV of the C–C peak in the XPS spectrum of MPA-capped CuInS_2_ QDs can be attributed to the stronger interaction of the bifunctional MPA ligand with the QD surface. Unlike TAA, MPA contains a polar –COOH group, which results in a small reduction in the C–C binding energy compared to other ligands. In contrast, TGA has a shorter chain, so the effect of surface coordination on the C–C peak is less pronounced, and TAA lacks a polar –COOH group altogether, making it essentially monofunctional and poorly bound, which also explains why no similar shift is observed for TAA.

Fourier transform infrared (FTIR) spectroscopy (Vertex 70V, Bruker, Billerica, MA, USA) was performed to analyze the functional groups attached to the CuInS_2_ QDs. The FTIR spectra of the different ligand-capped QDs are shown in Figure 2b. Prior to ligand exchange, the CuInS_2_-DDT QDs exhibit a strong C–H stretch between 3000 and 2900 cm^−1^, corresponding to alkane groups present in DDT. After ligand substitution, this peak intensity drops significantly, indicating the removal of long-chain ligands. Furthermore, new peaks emerge near 1700 cm^−1^ for the MPA- and TGA-treated samples, corresponding to C=O stretching vibrations from carboxylic acid groups, again confirming the successful replacement of the original ligands.

### 3.2. Optical Characteristics of the Ligand-Exchanged CuInS_2_ QDs

**Figure 3:**
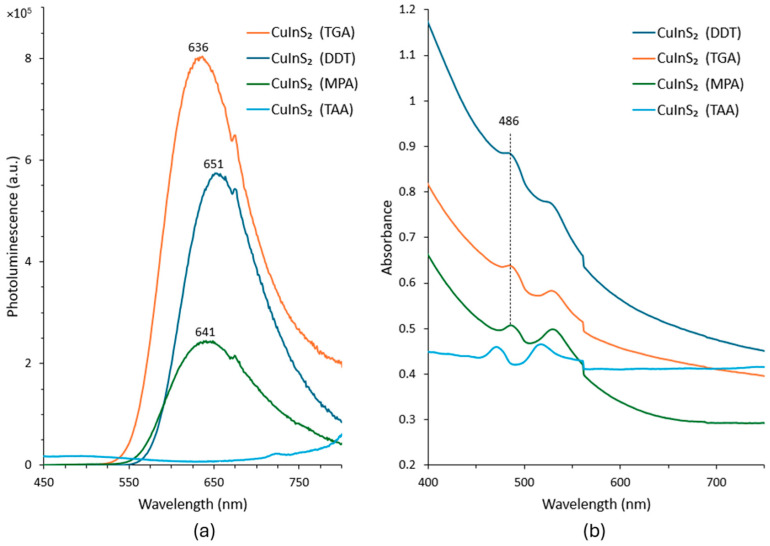
(**a**) Photoluminescence spectra, and (**b**) absorption spectra, of the synthesized CuInS_2_ QDs capped with DDT, TGA, MPA, and TAA ligands.

The optical characteristics of the CuInS_2_ QDs before and after ligand exchange were examined using photoluminescence (PL) and UV–visible absorption measurements. PL spectroscopy (Edinburg Instruments) with a 450 W Xenon lamp as the excitation light source was used to measure the PL emission of the QDs. The absorbance spectra were obtained using a PerkinElmer Lambda 1050 UV-visible spectrometer. Figure 3a presents the PL emission spectra of CuInS_2_ QDs capped with different ligands.

The characteristic peak for the QDs with DDT, MPA, and TGA capping is observable. As mentioned earlier, the TAA ligand exchange process resulted in poor dispersity; it also results in poor PL, likely due to the formation of surface defects. The increased defect formation in TAA-capped CIS QDs can be attributed to chemical surface etching and a lack of colloidal stabilization. Unlike bifunctional ligands like TGA and MPA, which utilize a polar –COOH group to maintain electrostatic repulsion and surface integrity, TAA consists of an inert methyl group attached to a –COSH group, making it essentially monofunctional in its interaction with the QD surface [34]. The absence of a strong polar functional group results in poor dispersion and significant aggregation. Furthermore, TAA can aggressively etch the QD surface [35], creating defect states that quench the PL. This suggests that TAA is not an effective ligand for CuInS_2_ QDs. The varying levels of PL peak intensity and also the slight variation in the peak positions can result from the exchange processes adopted for the different ligands, affecting the surface coverage yield, surface defect levels, and passivation. In fact the extent and success of ligand exchange depend on the binding configuration, coverage density, and interaction of the ligands with the surface atoms of the core QD. Localized trap states associated with the thiol-stabilizing ligands can affect the luminescence and quantum yield in QDs [36,37]. In terms of emission intensity, the TGA-capped QDs resulted in the highest PL intensity, indicating improved surface passivation. The absorption spectra for all four types of CuInS_2_ QDs are shown in Figure 3b. The primary absorption peak remains unchanged at ≈ 485 nm across the DDT-, MPA-, and TGA-terminated CuInS_2_ QDs samples. The apparent blue shift in the absorption peak of the TAA-capped CuInS_2_ QDs is attributed to surface modification induced by the TAA ligand exchange process. As mentioned earlier, TAA results in poor colloidal stability and incomplete surface passivation, which can lead to surface defect formation or partial surface etching, effectively reducing the core size of the QDs. This reduction can manifest as a blue shift in the absorption edge.

### 3.3. Electrical Characteristics of the CuInS_2_ QD Films

**Figure 4:**
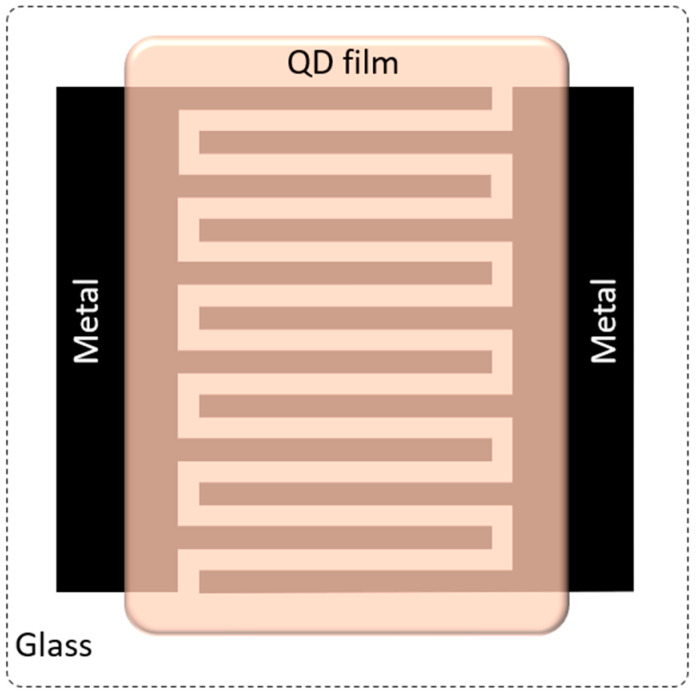
Schematic of the MSM test structure used for the electrical characterization of the QD films. Metal fingers are 20 μm in length with 3 μm spacing.

To evaluate the electrical performance of the QD films with different surface ligands, we fabricated planar MSM test structures: a 300 nm-thick aluminum film was e-beam evaporated onto RCA-cleaned glass substrates measuring 22 × 22 mm. An array of 40 test structures was then formed on each glass substrate by patterning the Al film using maskless photolithography (Heidelberg MLA 150) followed by metal etching. The QD film was then deposited onto the interdigitated areas by drop-casting method, while the electrode contact pads were kept protected. The drop-casting procedure used resulted in a 2 µm-thick film for all the different ligand-terminated CuInS_2_ QDs dispersed in ethanol. A schematic of a single test structure is depicted in Figure 4. The metal fingers were 20 μm in length with 3 μm spacing.

**Figure 5:**
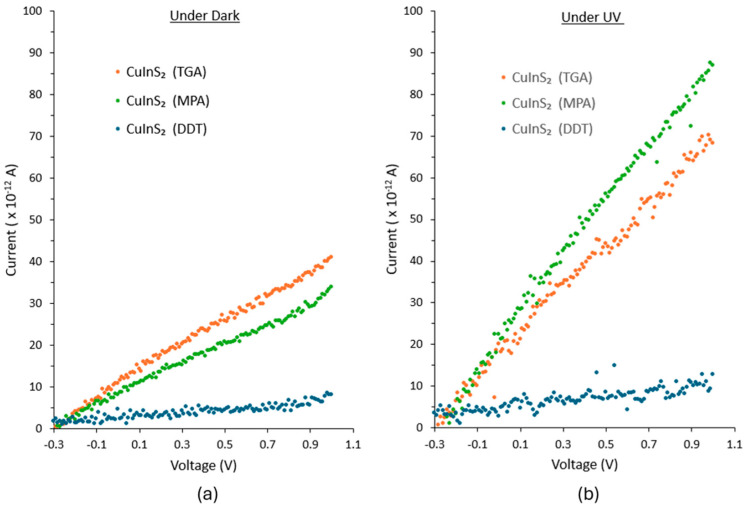
I-V characteristics of different ligand terminated CIS QDs (**a**) under dark and (**b**) under UV illumination.

Figure 5 shows the current-voltage (I-V) characteristics of the different ligand terminated CuInS_2_ QD films under both dark and UV illumination (Cole Parmer, 4 W 365 nm UV lamp). Table 1 shows the results for QD films capped with DDT, MPA, and TGA ligands. The results demonstrate that the current flow is strongly influenced by the choice of capping ligand for the CuInS_2_ QDs. Compared with QD films capped with the long-chain DDT ligand, those with shorter-chain ligands (MPA, TGA) exhibited approximately 4–5 times higher current in the dark and about 7–9 times higher current under UV illumination. This indicates the formation of efficient charge transport pathways as a result of the ligand exchange process. Ligand length directly controls the inter-dot spacing and therefore the degree of electronic coupling between neighboring CuInS_2_ QDs. For long-chain ligands, the large inter-dot separation significantly suppresses wavefunction overlap, leading to transport dominated by thermally activated carrier hopping between localized states. In this regime, the carrier hopping rate is exponentially dependent on inter-dot distance, resulting in low effective mobility and limited photoconductive response. With decreasing ligand length, the inter-dot spacing is reduced, which enhances wavefunction overlap and increases the electronic coupling energy between adjacent QDs. Sufficiently strong coupling can lead to the formation of delocalized minibands [38]. Furthermore, in QD field-effect transistors, higher field-effect mobilities have been observed in devices with shorter inter-dot spacings in the QD system [39]. From Table 1, the photocurrent-to-dark current ratios were 2.6 and 1.7 for MPA- and TGA-capped QD films, respectively. The higher ratio observed in the case of the MPA ligand, despite its slightly longer chain than that of TGA, is likely associated with differences in photocarrier extraction yield and/or recombination dynamics. This excellent photoresponse confirms the efficacy of the ligand exchange processes applied to the QDs post-synthesis.

**Figure 6:**
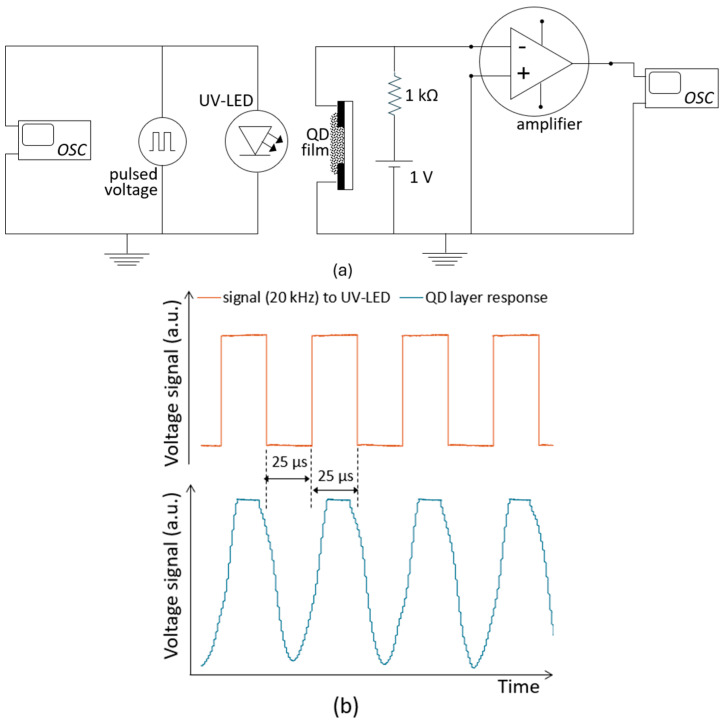
(**a**) Schematic of the measurement setup for the response of the QD film to pulsed UV illumination. (**b**) Input pulse to the UV LED (orange) and the photoresponse of the TGA-capped CuInS_2_ QD film (blue).

Considering the good photoresponse and strong photoluminescence characteristics, we tested the response of the TGA-capped MSM device to pulsed optical excitation. The measurement setup is illustrated in Figure 6a. A 20 kHz square-wave signal was applied to a UV LED. The resulting pulsed illumination from the LED was incident normal to the surface of the QD film in the MSM device. The device was biased with a 1-volt supply through a resistor, as shown in the figure. The voltage across the QD film, resulting from its response to the incident pulsed light, was recorded. Figure 6b shows the input voltage pulse to the UV-LED and the corresponding photoresponse of the QD-MSM device. The response of the ligand-exchanged CuInS_2_ film in a simple MSM structure indicates that this device is a good candidate to operate in photodetector mode.

Recent advancements in I–III–VI QD photodetectors have seen a significant push towards complex hybrid architectures and core/shell engineering to achieve enhanced performance. While these devices may have high photocurrent–dark current ratios and short response times, they are limited by intensive and costly multi-step processes [40,41,42,43]. In contrast, our lateral MSM device utilizes a scalable core-only synthesis process and simple device architecture, avoiding high expenses and multi-layer complexity of vertical stacks. Also, the primary objective of our work was to assess the influence of different ligands on the photoconductive behavior of CuInS_2_ QD films, rather than to benchmark a fully engineered photodetector.

## 4. Conclusions

In this work, we have synthesized CuInS_2_ quantum dots (QDs) stabilized with long-chain DDT ligands and subsequently performed ligand exchange using short-chain molecules, namely MPA, TGA and TAA, to improve the charge transport characteristics of the QD system. Successful ligand replacement was confirmed through XPS and FTIR analyses. Photoluminescence measurements revealed strong emission of the short-chain MPA and TGA-terminated QDs. TAA, despite its short chain length, appeared to introduce more surface defects upon exchange and also decrease the monodispersity of the colloidal solution. To evaluate the influence of ligands on the electrical conductivity of the QD films, we fabricated simple lateral MSM structures with different ligand-terminated QD films drop-cast onto interdigitated electrodes on glass substrates. Steady-state I-V measurements demonstrated that devices employing MPA- and TGA-capped films exhibited markedly improved charge transport and photocurrent compared to their original, DDT-capped counterpart. Transient response measurements further showed that the device responds well to pulsed UV illumination. These results highlight the suitability of MSM arrays employing low toxicity, ligand-exchanged CuInS_2_ QD films for optical switching and detection applications.

## Figures and Tables

**Table 1:** Measured current (under dark and UV illumination) at 1 V bias in the MSM test structures for QD films with different ligands.

Measured Current at 1 Volt Bias			
QD Film Type	Under Dark (pA)	Under UV (pA)	Ratio (Light/Dark)
CuInS_2_—MPA	33.2	87.4	2.6
CuInS_2_—TGA	40.6	68.9	1.7
CuInS_2_—DDT	8.0	9.3	1.2

## Data Availability

Data is contained within the article.
